# Geometry-dependent functional changes in iPSC-derived cardiomyocytes probed by functional imaging and RNA sequencing

**DOI:** 10.1371/journal.pone.0172671

**Published:** 2017-03-23

**Authors:** Christopher A. Werley, Miao-Ping Chien, Jellert Gaublomme, Karthik Shekhar, Vincent Butty, B. Alexander Yi, Joel M. Kralj, Blox Bloxham, Laurie A. Boyer, Aviv Regev, Adam E. Cohen

**Affiliations:** 1 Department of Chemistry and Chemical Biology, Harvard University, Cambridge, Massachusetts, United States of America; 2 Broad Institute, Cambridge, Massachusetts, United States of America; 3 Department of Biology, MIT, Cambridge, Massachusetts, United States of America; 4 Department of Biological Engineering, MIT, Cambridge, Massachusetts, United States of America; 5 David H. Koch Institute for Integrative Cancer Research, MIT, Cambridge, Massachusetts, United States of America; 6 Howard Hughes Medical Institute, Cambridge, Massachusetts, United States of America; 7 Department of Physics, Harvard University, Cambridge, Massachusetts, United States of America; Centro Cardiologico Monzino, ITALY

## Abstract

Human induced pluripotent stem cell-derived cardiomyocytes (hiPSC-CM) are a promising platform for cardiac studies *in vitro*, and possibly for tissue repair in humans. However, hiPSC-CM cells tend to retain morphology, metabolism, patterns of gene expression, and electrophysiology similar to that of embryonic cardiomyocytes. We grew hiPSC-CM in patterned islands of different sizes and shapes, and measured the effect of island geometry on action potential waveform and calcium dynamics using optical recordings of voltage and calcium from 970 islands of different sizes. hiPSC-CM in larger islands showed electrical and calcium dynamics indicative of greater functional maturity. We then compared transcriptional signatures of the small and large islands against a developmental time course of cardiac differentiation. Although island size had little effect on expression of most genes whose levels differed between hiPSC-CM and adult primary CM, we identified a subset of genes for which island size drove the majority (58%) of the changes associated with functional maturation. Finally, we patterned hiPSC-CM on islands with a variety of shapes to probe the relative contributions of soluble factors, electrical coupling, and direct cell-cell contacts to the functional maturation. Collectively, our data show that optical electrophysiology is a powerful tool for assaying hiPSC-CM maturation, and that island size powerfully drives activation of a subset of genes involved in cardiac maturation.

## Introduction

Human iPSC-derived cardiomyocytes (hiPSC-CM) are morphologically [1], genetically [2], and functionally closer to embryonic than adult primary myocytes [3]. Upon transplantation into rodent or pig heart, hiPSC-CM take on more mature attributes [4,5], implying that these cells are capable of maturation. Mature primary cardiomyocytes dissociated and maintained in culture rapidly lose their characteristic morphology and take a more immature appearance [6]. Thus the cell culture environment favors an immature phenotype, as measured by morphology, gene expression, and electrophysiology.

Significant effort has been devoted to finding protocols that enhance maturation (see, e.g. [7–9]) by mimicking as closely as possible the *in vivo* milieu. Soluble factors [10–12], substrate mechanical stiffness [13], and electrical and mechanical pacing [14,15] all play an important role. Cell-cell contacts may also mediate local signaling mechanisms that drive maturation. Cell patterning is a powerful tool for dissecting the role of different factors in maturation and development [16,17]. Cardiomyocytes grown on grooved substrates showed enhanced Ca^2+^ oscillations [18], and cardiomyocytes grown on narrow strips showed enhanced sarcomere alignment [19]. By tuning the geometry of cellular islands, one can probe the nature of intercellular interactions that influence development.

In assaying functional maturity, tools of optical electrophysiology are particularly useful because the electrical and calcium dynamics reflect the aggregate activity of a large number of transporters, pumps, and ion channels [20]. Recently described systems enabled simultaneous voltage and calcium imaging in free-running or electrically paced hiPSC-CM [21], optogenetic pacing and either voltage or calcium imaging [22], or optogenetic pacing with simultaneous voltage and calcium imaging [23].

We applied simultaneous voltage and calcium imaging using a genetically encoded dual-function reporter, CaViar [23,24], to probe the functional maturation of hiPSC-CM grown on micro-fabricated islands of different sizes and shapes. HiPSC-CM grown on larger islands showed greater functional maturity by several measures of voltage and Ca^2+^ dynamics. Comparison to numerical simulations based on the Aliev Panfilov model [25] established that at typical cell densities, hiPSC-CM cultures are in the strong electrotonic coupling limit, i.e. that the action potential waveform at each cell is dominated by the collective activity of its neighbors, not by its own complement of ion channels.

To explore the underlying mechanism for island size-dependent maturation, we performed RNA sequencing on hiPSC-CM grown on small or large islands. There were small but statistically significant differences in gene expression between small and large island cultures. Remarkably, for a subset of genes, island size-dependent changes explained ~58% of the difference between cardiac progenitors and adult cardiomyocytes. Thus, within the global set of transcriptional changes that accompany cardiac maturation, there is a small subset of genes that is highly sensitive to island size, whereas most genes are not.

Finally, we grew hiPSC-CM on islands of different shapes designed to probe the relative contributions of paracrine factors, electrical coupling, and direct cell-cell contacts to island size-dependent maturation. We found that nearest-neighbor contact interactions played the most important role in driving functional maturation. Our results demonstrate that optical electrophysiology measurements provide robust, moderate-throughput functional characterization of hiPSC-CM that can be combined with transcriptional profiling to identify key factors that regulate maturation of hiPSC-CM.

## Materials and methods

### Cell culture substrates

Cell patterning was performed in glass-bottom dishes (In Vitro Scientific, D35-20-1.5-N). Dish surfaces were cleaned with 5 min. air plasma (SPI Plasma-Prep II) to expose the SiOH groups. The glass part of the dish was treated with silane solution comprising 0.5% vol/vol 3-(trimethoxysilyl)propyl methacrylate (Sigma 440159), 2% glacial acetic acid, and 97.5% anhydrous ethanol (200 μL). The dish was incubated for 30 min in an oxygen-free N_2_ atmosphere, and rinsed 3 times with absolute ethanol. The dish was baked for 30 min at 65°C in a vacuum oven or N_2_-purged glove box to complete covalent bonding between the trimethoxysilyl groups and the hydroxyl from the glass, with the evolution of methanol.

Next we polymerized a polyacrylamide gel on the functionalized glass surface, leading to covalent bonding of the methacrylate to the functionalized glass. As in Ref [26], the gel thickness (typically 40 μm) and surface flatness were controlled by performing the polymerization between the activated glass and a siliconized, non-stick coverslip (Hamilton Research, HR3-239). Siliconized coverslips were cleaned prior to use by 3 min. sonication in ultrasonic cleaning solution (Fisher 15-335-80) followed by a 4x rinse in deionized water.

The polymerization solution was 8% W/V acrylamide (Sigma A4058), 0.2% W/V bis-acrylamide (Sigma M1533), 0.1% V/V TEMED (Sigma T7024), 1.2 mg/mL potassium persulfate (KPS; Sigma 216224), and 4.2 mg/mL acryl-NHS (Sigma A8060). To slow hydrolysis of the highly reactive NHS groups, we performed the polymerization in a 40 mM phosphate buffer at pH 7, which extended the NHS lifetime to about 1 hour at room temperature. The mixture was aliquoted so addition of the radical initiators TEMED and KPS, brought the volume to 1 mL. 1 μL of TEMED was added, the solution was stirred on a vortexer, and then 100 μL of KPS (12 mg/mL) was added and stirred on a vortexer. 10 μL of the solution was pipetted onto the center of each of several glass-bottomed dishes, followed immediately by placing the siliconized coverslip on top. Radical-initiated polymerization began after the oxygen from the solution confined between the two glass plates was consumed.

After polymerization, the top coverslip was pried off using a sewing needle, and the gel was rinsed three times with phosphate buffer, pH 7, and dried under nitrogen. For long-term storage, we wrapped with aluminum foil to protect from light, vacuum-sealed with desiccant (FoodSaver 2840), and stored at -80°C. The dishes were usable for several months with no detectable decline in performance.

### Patterned adhesion for patterned cell growth

To grow cells on islands of pre-determined size and shape, standard soft lithography techniques [27] were used to make patterned islands of fibronectin covalently bound to the polyacrylamide. In brief, patterns were designed in AutoCAD and printed on a mylar transparency (CAD Art Services). The pattern was transferred to a layer of SU-8 3025 on a Si wafer via contact lithography, followed by removal of un-exposed photoresist with SU-8 developer. The SU-8 master was then used as a template for casting a poly(dimethylsiloxane) (PDMS) stamp. The PDMS stamp was exposed to a solution of fibronectin (Yo Proteins #663, 0.05 mg/mL) for 30 min following by aspiration and drying in air for 10 min. The pattern was then printed onto the NHS-functionalized acrylamide. The NHS covalently bonded to the fibronectin, forming a stable cell-adherent pattern. Fibronectin fluorescently labeled with green dye for testing cell patterning was obtained from Cytoskeleton, Inc. (FNR02-A). In preparing fibronectin solutions one must take care to ensure the Tris or other buffers containing primary amines are rigorously excluded from the solution.

### Cloning of lentiviral CaViar construct

The CaViar construct was cloned into the pLenti-CMV-puro plasmid (addgene #17448). This backbone is a third generation lentiviral plamsid with puromycin resistance that drives transgene expression under a CMV promoter. The plasmid was cut with PmeI and SalI and purified by gel elution.

The CaViar construct is a fusion of v5 epitope tag, QuasAr2, the Kir2.1 trafficking sequence (TS), and GCaMP6f. The Kir2.1 TS has been used with both QuasAr2 and CheRiff to improve rhodopsin localization to the plasma membrane. The construct was generated by PCR amplification of both QuasAr2 (with the epitope tag), and GCaMP6f (with the TS). The PCR primers also included overlap regions to the plasmid backbone necessary for isothermal assembly. The entire plasmid was generated by a 3 component isothermal reaction at 50 C for 1 hour. The final construct was checked by sequencing.

### Cardiomyocyte plating and maintenance

HiPS-CM (iCell cardiomyocytes), plating and maintenance medium were purchased from Cellular Dynamics International. Cells were thawed and cultured following manufacturer instructions, and maintained at 37°C with 5% CO_2_. Cells were plated at a density of 50k/cm^2^, calibrated by a Trypan blue stain. Cells were plated initially in just the recessed region of the glass-bottomed dish. After 1 hour, a very gentle rinsing removed dead and un-adhered cells and the volume of plating medium was increased to 1 mL. 48 hours after plating, the plating medium was aspirated and replaced with maintenance medium. While performing the medium exchange, extra care is required to prevent the patterned cardiomyocytes from lifting off the dish. The medium was exchanged every other day after plating.

### Production of lentivirus and infection of cardiomyocytes

Lentivirus was prepared in HEK cells following established protocols [28]. In brief, low passage number HEK293T cells were plated onto gelatin-coated (Stemcell technologies, #07903) 15-cm dishes. To ensure uniform culture density, pelleted cells were carefully broken up and re-suspended in 16 mL of DMEM + 10% FBS (DMEM10) before plating. Cells were grown on well-leveled incubator shelves to ensure uniform growth conditions across the dish. When HEK cells reached 80% confluence, the medium was exchanged to a serum-free medium. After 1–2 hours, cells were transfected using polyethylenimine (PEI; Sigma 408727). Into 1.2 mL of DMEM were added 14 μg of the vector plasmid, 9 μg of the 2^nd^ generation packaging plasmid psPAX2 (Addgene #12260), and 4 μg of viral entry protein VSV-G plasmid pMD2.G (Addgene #12259). The mixture was vortexed with 36 μL of 1 mg/mL PEI, incubated for 10 min, and added dropwise to the plate. After 4 hours, the medium was exchanged back to 16 mL DMEM10 to avoid PEI toxicity. Typically 50% of cells expressed the fluorescent reporters 24 hours post transfection and 100% of cells expressed at 48 hours. The supernatant was harvested at 48 hours post transfection, centrifuged 5 min at 500g to pellet cells and debris, and filtered through a 0.45 μm filter. The un-concentrated virus was aliquoted and stored at -80°C for later use. Induced pluripotent stem cell (iPSC)-derived cardiomyocytes were purchased from Cellular Dynamics International (CDI). According to manufacturer specifications, the majority of the cells (~95%) showed a ventricular phenotype. Cardiomyocytes were infected with Optopatch constructs 10 days after plating by adding 100 μL of the lentivirus and imaged 4 days post viral infection. The cardiomyocyte medium was replaced with fresh maintenance medium 24 hours after adding the virus.

### Microscope

Experiments were conducted on a home-built inverted fluorescence microscope described in Ref. [29]. The microscope comprised a custom optical system developed around a high numerical aperture, low magnification objective (2x, numerical aperture 0.5). Briefly, illumination for voltage imaging (QuasAr2 fluorescence) was provided by six lasers at 635 nm, 500 mW (Dragon Lasers 635M500), combined in three groups of two. Illumination was coupled into the sample from below, without passing through the objective, using a custom fused silica prism near the critical angle for total internal reflection at the glass-water interface. Sample fluorescence passed through the custom prism and was collected by a low-magnification objective (Olympus 2x MVX Plan Apochromat, numerical aperture 0.5), passed through an emission filter, and imaged onto a scientific CMOS camera (Hamamatsu Orca Flash 4.0). This microscope imaged a 1.2 x 3.3 mm field of view with 3.25 μm spatial resolution and 2 ms temporal resolution. Blue illumination for GCaMP6 imaging was provided by a 473 nm, 1 W laser (Dragon Lasers), modulated in intensity by an acousto-optic modulator and modulated spatially by a digital micromirror device (DMD, Digital Light Innovations DLi4130 –ALP HS). The DMD was re-imaged onto the sample via the 2x objective. CaViar (GCaMP6 fused to QuasAr2) imaging was performed at a blue laser intensity of 0.12 W/cm^2^ with a 10 ms exposure time and a red laser intensity of 33 W/cm^2^ with a 4 ms exposure time. Each movie comprised 5000 frames of voltage imaging (20 s), immediately followed by 2000 frames of Ca^2+^ imaging (20 s).

### CaViar imaging

Cells were imaged after 10–14 days in culture. Shortly before imaging, the medium was replaced with a low-autofluorescence imaging buffer and allowed to equilibrate for 30–60 min (37°C, 5% CO_2_). The imaging buffer matched the osmolarity and ion concentrations of the maintenance medium, but lacked phenol red, vitamins, and amino acids. The formula in mM was: 1.8 CaCl_2_, 2.5x10^-4^ Fe(NO_3_)_3_, 0.81 MgSO_4_, 5.3 KCl, 44 NaHCO_3_, 129 NaCl, 0.91 NaH_2_PO_4_, 1 sodium pyruvate, 10 D-(+)-galactose. Imaging on the microscope was performed at 37°C under 5% CO_2_ using a home-built environmental chamber.

### Fluorescence analysis

Analysis was performed using custom Matlab software. Islands with incomplete cell coverage or islands that did not show beating in both calcium and voltage were excluded from the analysis. For each island, a fluorescence trace was calculated from the mean fluorescence over the island, and a background trace was calculated by averaging over a cell-free region surrounding each island. The background was subtracted from the island signal. Fluorescence traces were corrected for photobleaching, but otherwise not filtered. To increase the signal-to-noise ratio, each pixel’s time-trace was correlated with the average fluorescence trace, and more strongly-correlated pixels were weighted more strongly when re-calculating the average, as in [30]. Fluorescence traces were normalized to the baseline. The pixel re-weighting mitigated the effects of bright fluorescent aggregates that were insensitive to voltage or calcium. Voltage and calcium spikes were detected in each time trace using an edge-detection algorithm, and waveform shape characteristics including spike amplitude, duration, and rise time were extracted by calculating the times at which the waveform crossed user-specified thresholds. All fluorescence recordings were analyzed using the same parameters with no user intervention.

### Numerical simulations

Numerical simulations of action potential dynamics were performed for islands comprised of a rectangular array of electrically coupled cells, each with its own spontaneous beat frequency. Each cell was described by the Aliev-Panfilov model [25]. Our simulations used the equations:

$$\frac{du_{ij}}{dt}=ku_{ij}\left(1-u_{ij}\right)\left(u_{ij}-a\right)-u_{ij}v_{ij}+c_{ij}+\Phi \left(u_{i-1j}+u_{i+1j}+u_{ij-1}+u_{ij+1}-4u_{ij}\right)$$

$$\frac{dv_{ij}}{dt}=\varepsilon \left(u_{ij}\right)\left(ku_{ij}-v_{ij}\right)$$

where ε(*u* < 0.05) = 1 and ε(*u* > 0.05) = 0.1, and where *k* is a rate constant, *u*_*ij*_ is the excitation parameter (analogous to voltage), *c*_*ij*_ is a drive current, Φ is the nearest-neighbor coupling constant, and *v*_*ij*_ is an internal recovery parameter. The indices *i*, *j*, give the row and column coordinates of each cell. To match the data on conduction velocity, we set Φ ≈ 400. To produce realistic action potential waveforms we set *k* = 10 and *a* = 0.05. To match the mean beat rate we set the mean leak current per cell to *c*_mean_ = 0.09. To capture cell-to-cell variability, the leak current in each cell was sampled from a Gaussian distribution with a standard deviation of 22%. Non-absorbing boundary conditions were used. The systems of differential equations were solved using *MatLab*’s ode23 solver, which implements the Bogacki–Shampine method[31], over *t* = [0, 120].

Island sizes of 1x1, 2x2, 4x4, 8x8, 12x12, and 16x16 cells were simulated. The largest islands corresponded, approximately, to a 1x1 mm island of hiPSC-CM. Each island size was simulated 52 times with values of *c*_*ij*_ resampled between trials. For each island and each simulation, we calculated an intensity trace, *I*(*t*) = 〈*u*_*ij*_(*t*)〉_*ij*_. Peaks in *I*(*t*) were identified and the interval between the last two peaks was used to determine the beat period. The mean and standard deviation in beat period were calculated for each island size.

### RNA sequencing

Libraries were prepared using the SMART-seq2 protocol [32] modified to use Maxima H Minus enzyme for reverse transcription (Thermo Scientific) [33] as follows: population controls were generated by extracting total RNA using a RNeasy plus Micro RNA kit (Qiagen) according to manufacturer’s recommendations, and 1 μL of RNA in water was added to 3 μL of elution mix. Amplified cDNA products were purified using AMPure XP SPRI beads (Beckman Coulter) and eluted in TE buffer (Teknova). Cleaned-up amplified cDNA was used for library construction using the Nextera XT DNA Sample Preparation Kit and Nextera XT Index Kit (Illumina). Libraries were then pooled and cleaned up using AMPure XP SPRI beads (Beckman Coulter Genomics). Final library quality and quantity were assessed using a DNA High Sensitivity chip (Agilent).

### Analysis of RNA sequencing data

We prepared 21 RNA-seq libraries (11 large islands and 10 small islands). Libraries were sequenced to an average depth of 568,867 paired-end reads of length 25 bp. The reads were mapped to the UCSC human transcriptome (genome build hg19) using RSEM v1.2.19 and Bowtie v1.1.1 as the read mapper [34] and for all genes, read counts and transcript-per-million (TPM) were retrieved [35]. On average 93% of the reads mapped to the genome in every sample (range 91.8–94.6%), whereas 63% of the reads mapped to the transcriptome (range 53.5–71.8%). TPM results were log-transformed after the addition of 1 to avoid zeros.

We calculated for each of the 10 small island libraries and 11 large island libraries the number of genes detected at TPM > 10. The average number of genes detected per sample was 6000±200 (range 5722–6386), confirming that all libraries were of sufficient complexity to be retained for further analysis. We then excluded all genes whose average TPM in both the small island samples and the large island samples (calculated separately) was less than 10, retaining 6328 genes that were appreciably expressed for further analysis. Changing this threshold by 30% (lower or higher) added or subtracted the number of genes by less than 10% (S1 Table).

Differential expression analysis was performed using the R package DESeq2 [36] with the RSEM-inferred matrix of read counts as input, containing only the 6328 appreciably expressed genes. DESeq2 first normalizes the raw counts to adjust for differences in library size across replicates, and uses a statistical framework that models the normalized read counts for each gene as a negative binomial distribution to find statistically significant differences between the two conditions. DESeq2 output a list of differentially expressed genes ranked by the average log2-fold change in expression between the large and the small islands (“effect size”), and also an FDR adjusted p-value for each gene (“statistical significance”).

Gene Ontology (GO) terms were evaluated corresponding to “Process”, “Function” and “Cellular component”, for the differentially expressed genes in each group using GOrilla [37]. The appreciably expressed 6328 genes were used as the background set for the purpose of calculating enrichments using the hypergeometric test.

#### Comparison with transcriptomic datasets

Existing transcriptional data from a human embryonic stem cell (ESC) to cardiomyocyte (CM) differentiation time-course were obtained from [38]. Data sets can be accessed under GEO GSE53567. The reads were mapped and quantified using RSEM v. 1.2.15 [35] and Bowtie 1.0.1 against hg19, ENSEMBL annotation 68, and transcripts per million (TPM) were retrieved from the output. Human left and right ventricular (E095 and E105) and right atrial (E104) samples were retrieved from the NIH Epigenomics Roadmap project (http://egg2.wustl.edu/roadmap/web_portal/), and RPKM values were converted into TPMs by summing RPKMs over all protein-coding and non-coding transcripts, and dividing individual genes’ RPKM values by the sum, for each sample individually. Genes were merged based on their ENSEMBL IDs for the Roadmap and ESC2CM datasets, and merged further with the samples from the present study based on gene symbols, resulting in a set of 19836 genes. For further analysis we selected a subset of 5831 genes with an average TPM greater or equal to 10 in either of the small or large island groups as the background. Z-scores were calculated across all samples by subtracting a gene’s mean TPM value from each sample, and dividing by the standard deviation.

## Results

### Cell patterning

We adapted a published cell patterning technique [27] to accommodate the demands of long-term cell culture and the strong traction forces from beating cardiomyocytes (Methods, S1 Fig). To minimize stress at the cell-substrate interface and to facilitate cell patterning, we grew hiPSC-CM on a compliant polyacrylamide substrate [39], doped with acryl-NHS. Patterns of fibronectin were transferred to the gel surface via a poly(dimethylsiloxane) (PDMS) stamp, whereupon covalent reaction with the NHS irreversibly bonded the fibronectin to the gel. The bare polyacrylamide between the islands was highly cytophobic, an important attribute because even a single cell bridging two islands could introduce spurious electrical coupling.

**Fig 1:**
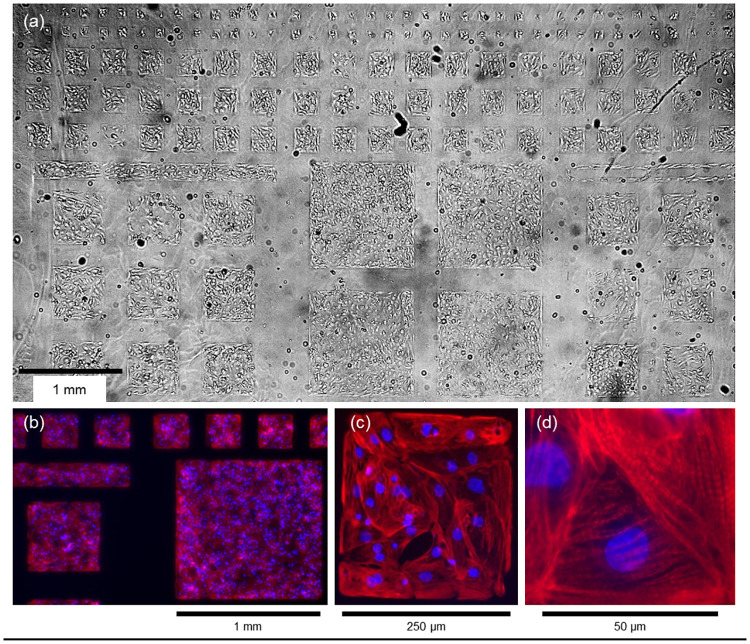
Patterned cardiomyocytes. Cardiomyocytes were patterned on a cytophobic polyacrylamide surface patterned with covalently bound fibronectin by micro-contact printing. a) Trans-illumination image of patterned cardiomyocytes showing the full 6 mm field of view. b) Fluorescence images of cardiomyocytes on patterned islands with nuclear stain DAPI (blue) and myofibril stain cardiac troponin T (red). c) A magnified view of one 250 μm island. d) Myofibril banding is clearly visible at single-cell resolution.

The stamping yielded extensive, high-quality fibronectin patterns as shown with fluorescently labelled fibronectin (S1 Fig). Cells grew throughout the fibronectin-coated regions, and were absent on the bare polyacrylamide both for HEK 293T cells (S1 Fig) and for hiPSC-CMs (Fig 1). Covalent bonding of the fibronectin to the gel was necessary for long-term cell culture; otherwise cellular traction forces gradually delaminated the fibronectin, leading to retraction of the island edges or complete island loss. hiPSC-CMs growing on the islands contracted vigorously and spontaneously, consistent with myofibril banding revealed by immunostaining with cardiac troponin T (Fig 1 & S10 Fig).

### Functional mapping

To perform voltage and Ca^2+^ imaging over a wide field of view, we used a custom optical system developed around a 2x NA 0.5 objective (Olympus MVPLAPO 2 XC) [29]. This system imaged a 6 x 6 mm field of view with 3.25 μm spatial resolution and 100 Hz frame-rate. To achieve 500 Hz frame-rates for voltage imaging, the field of view was limited to 1.2 x 3.3 mm, enabling parallel single cell-resolved measurements on approximately 1,500 hiPSC-CM.

We seeded hiPSC-CM on square islands of edge length 50, 100, 250, 500 and 1000 μm and in contiguous cultures. Mean cell diameter was 50 μm. The approximate percentages of cells on the perimeter as a function of island size were: 100% for 50 μm and 100 μm, 64% for 250 μm, 36% for 500 μm, and 19% for 1000 μm islands. Thus the island sizes spanned perimeter-to-interior ratios ranging from mostly exterior to mostly interior. Cells began to beat spontaneously at 4 days post-plating (dpp). At 10 dpp we infected the cells with lentivirus containing the CaViar gene, comprised of a fusion of the voltage indicator QuasAr2 [29] and the calcium indicator GCaMP6f [40]. This construct was previously validated in hiPSC-CM, where it was shown to accurately reflect voltage and calcium waveforms[23]. Measurements were then performed at 14 dpp.

With the ultra-wide-field imaging system, a single 1.2 x 3.3 mm field of view contained up to 96 of the 50 μm islands or two of the 1 mm islands. Cells showed robust voltage and calcium transients with typical (geometry-dependent) magnitudes of ~180% ΔF/F for Ca^2+^ and ~35% ΔF/F for voltage. QuasAr2 waveforms were indistinguishable from simultaneous recordings using a fast voltage-sensitive dye (FluoVolt; S2 Fig). Action potential waveforms reported via FluoVolt fluorescence were indistinguishable between hiPSC-CM expressing QuasAr2 and those not expressing QuasAr2 (S2 Fig). These experiments confirmed that the QuasAr2 voltage indicator was a faithful and non-perturbative reporter of cardiac membrane voltage.

We developed an analysis pipeline to convert raw fluorescence movies to single-island fluorescence traces (S3 Fig). In brief, movies were first corrected for inhomogeneous background by subtracting the fluorescence in a boundary region around each island. The fluorescence of each island was calculated as a simple average over on-island pixels. The baseline fluorescence was calculated by a sliding minimum filter with a window longer than the beat period. Drift in baseline fluorescence was dominated by photobleaching of background autofluorescence rather than by photobleaching of reporters, so this background was subtracted from the single-island traces.

**Fig 2:**
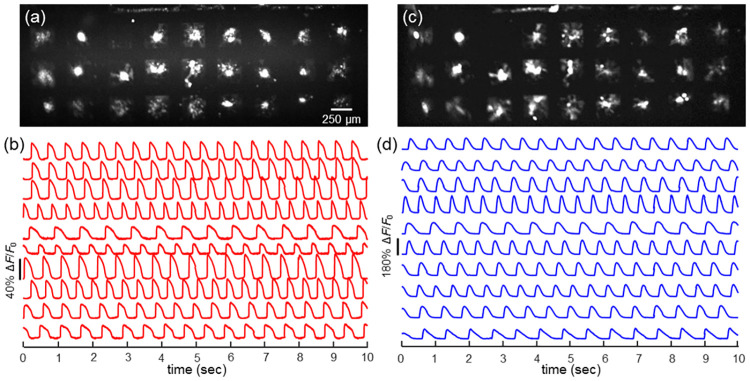
Optical electrophysiology recordings. (a) Fluorescence image of QuasAr2 expressed in 250 μm islands, all of which were monitored simultaneously at a 500 Hz frame rate. b) Fluorescence recordings of QuasAr2, indicating voltage, from islands in (a). The individual islands showed heterogeneity in spontaneous beat rate and action-potential waveform. c) Fluorescence of GCaMP6F expressed in the same islands as in (a). d) Fluorescence recordings of GCaMP6F, indicating Ca^2+^, from islands in (c). Data in (b) and (d) has been corrected for slow photobleaching, but not otherwise filtered.

Fig 2 shows QuasAr2 and GCaMP6F fluorescence images and corresponding intensity traces for 250 μm islands; the associated videos are in S1 and S2 Movies. Although some of the smallest islands lifted off the dish, the majority of islands remained adhered and beat regularly. The numbers of islands of each size used in analysis were: *n* = 65 (50 μm), *n* = 322 (100 μm), *n* = 211 (250 μm), *n* = 57 (500 μm), and *n* = 31 (1 mm). Measurement of these large datasets was necessary to achieve statistically significant results in the presence of large heterogeneity in the individual islands. This throughput was only possible due to the ultra-wide-field imaging system combined with sensitive fluorescent reporters.

The large quantities of optical electrophysiology data necessitated automated processing to extract action potential parameters. S4 and S5 Figs show the automatically parameterized voltage traces from islands of each size tested. From each island, we quantified peak times and pulse widths at 30%, 50%, and 70% of return to baseline for both voltage and calcium. For voltage we calculated the maximum upstroke rate, max(Δ*F*/*F*_0_/*dt*), and for calcium we calculated the upstroke time from 30% to 70% max fluorescence. For each island size we calculated a histogram of the Δ*F*/*F*_0_ values over all pixels (S6 Fig) and then calculated the mean of Δ*F*/*F*_0_ from all pixels that showed beating. To test for possible artifacts introduced by the analysis, we numerically subdivided movies of 1 mm islands into sub-regions whose size matched each of the smaller island sizes, and analyzed these as though they were smaller islands (S7 Fig). This subdivision did not change the calculated waveform parameters, confirming that the analysis was unbiased with respect to island size.

Each dish contained islands of all sizes, and all island sizes were measured on the same day. These in-dish comparisons removed errors from uncontrolled variability in culture conditions, seeding density, or subtle dish-to-dish variations in pH or temperature.

### Island size affects action potential parameters

**Fig 3:**
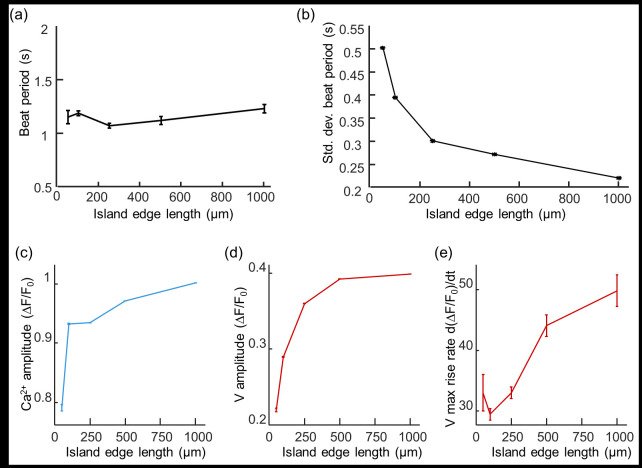
Island size-dependent action potential parameters in hiPSC-CM. a) Mean spontaneous beat period as a function of island size at 14 dpp. b) Standard deviation of beat period across islands, as a function of island size. c) Amplitude of the calcium transient as a function of island size. d) Amplitude of the voltage transient as a function of island size. e) Rise time (20% to 80%) of the voltage transient as a function of island size. Error bars in a,c-e represent s.e.m.

The mean spontaneous beat rate was largely independent of island size between 50 μm and 1000 μm (Fig 3a), but the island-to-island variability in spontaneous beat rate decreased as a function of island size (Fig 3b). As island size increased, we observed increases in the amplitudes of the Ca^2+^ and voltage transients (Fig 3c and 3d), and the upstroke velocity of the voltage (Fig 3e). In comparison to the 50 μm islands, in the 1 mm islands Ca^2+^ transient amplitudes were 1.23-fold larger (p = 3.3×10^-16^), voltage amplitudes were 1.81-fold larger (p = 1.1×10^−16^), and the upstroke velocity of the voltage was 1.51-fold faster (p = 0.0057, two-sided t-test for each). These parameters have been associated with increased functional maturity [3], which we examine in detail below. We did not detect island size-dependent changes in action potential width.

To interpret these size-dependent changes in hiPSC-CM function, we considered several possible models. HiPSC-CM have been reported to comprise a mixture of cells with atrial, ventricular, and nodal or pacemaker, phenotypes[41]. We reasoned that the pacemakers might have a heterogeneous distribution of intrinsic rates, and that the fastest pacemaker on each island might set the rate. In this ‘fastest cell’ model, one would expect larger islands to be more likely to contain an anomalously fast pacemaker. Thus the ‘fastest cell’ model predicted that larger islands would beat faster, simply as a consequence of extreme-value statistics when sampling from a heterogeneous distribution of pacemaker rates[42]. An alternative model was that island size directly affected the electrophysiology of the cells. Edge effects or differences in perimeter-to-area ratio between small and large islands might drive changes in ion channel function or patterns of gene expression.

To test the ‘fastest cell’ model quantitatively, we performed numerical simulations to study how island size affected action potential dynamics in the case where the individual cells were heterogeneous, but where the underlying statistical distribution of cellular properties was independent of island size. A great many cardiac numerical models have been developed, including models tuned specifically for hiPSC-CM[43]. We were interested in how the interactions of large numbers of hiPSC-CM affected the collective dynamics. For this type of question, it was not necessary to consider the individual ionic currents within each cell. Rather, it was sufficient to adopt a coarse-grained model which represented each cell as a few-parameter nonlinear oscillator. Of the models in this class, the phenomenological Aliev-Panfilov model was selected because it recapitulates many of the collective phenomena observed in real cardiac tissue (Methods)[25].

We fit parameters of the Aliev-Panfilov model to experimental measurements on cultured hiPSC-CM. In confluent monolayers, action potentials propagated with conduction velocity *v* = 120 mm/s and AP wavelength λ = 32 mm (S8 Fig). The largest island size (1 mm) was much smaller than the AP wavelength, implying that electrical influences spanned even the largest islands on a timescale short compared to the AP duration. To match the data on conduction velocity required a dimensionless coupling parameter in the Aliev-Panfilov model, Φ ≈ 400, corresponding to the strong electrotonic coupling limit. Other parameters in the model were selected to match the experimentally observed AP waveforms (S8 Fig). Each cell was given a static leakage current sampled from a Gaussian distribution, which led to a distinct beat rate in the absence of cell-cell coupling (S9a Fig).

In simulated islands comprised of strongly coupled cells, all cells beat synchronously and with indistinguishable AP waveforms, despite the heterogeneity in cell-autonomous properties. This observation implies that one would not expect to observe distinct atrial, ventricular, and nodal waveforms at the single-cell level, even if cells with corresponding ion channel compositions existed in the syncytium [44]. We did not observe distinct cell type-specific waveforms in the experimental data.

As in our experimental observations, the simulated beat rate was independent of island size, and the island-to-island standard deviation in the beat rate decreased as a function of island size, contrary to the expectations of the ‘fastest cell’ model. Similar results were obtained with a wide range of model parameters. Taken together, the simulations and data favor a model in which the spontaneous activity of the islands comes from the strongly coupled activity of many spontaneously active cells, rather than a model in which a few pacemakers drive a non-autonomous population. Furthermore, the ‘fastest cell’ model failed to account for the size-dependent trends observed in beat rate, beat rate variability, and action potential parameters.

### Island size affects gene expression profile

The striking island size-dependent trend in waveform parameters suggested that there might be functional differences between the individual cells grown on islands of different sizes. We sought a transcriptional signature of this trend. We made dishes with islands of 100 μm diameter (“small islands”), and matching dishes with confluent culture. Optical electrophysiology parameters were similar between the 500 μm and 1 mm islands (Fig 3), supporting the use of a confluent culture to explore the large-island limit. We used RNA sequencing to profile the transcriptional state of the cells in both conditions. We tested *n* = 10 biological replicates of the small islands and *n* = 11 replicates of the confluent culture. Total RNA was extracted from each replicate, and whole-transcriptome libraries were prepared using a modified version of the SMART-Seq2 protocol [32,33], and sequenced on the Illumina platform. Expression levels were quantified as log_2_ TPM (transcripts per million). A total of 5,831 genes yielded sufficient expression across samples for further analysis.

**Fig 4:**
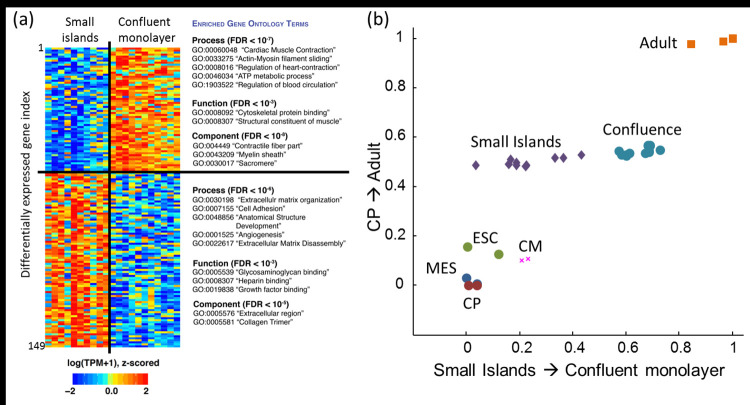
RNA sequencing to probe the difference between hiPSC-CM grown in small (100 μm) islands vs. a confluent monolayer. a) Confluent cells had greater expression of cardiac-related genes, while cells grown on small islands had greater expression of genes related to extracellular matrix, adhesion, and angiogenesis. b) Gene expression differences between small islands and confluent monolayer, projected onto two vectors in gene-expression space. The CP → Adult axis comprises the 3,463 genes that were significantly differentially expressed between cardiac progenitors and adult cardiomyocytes. The Small Islands → Confluent monolayer axis comprises the 149 genes that were significantly differentially expressed between the two island sizes. ESC: embryonic stem cells MES: mesoderm, CP: cardiac progenitors, CM: embryonic stem cell-derived cardiomyocytes.

Analysis of differential expression between large and small islands (see Experimental Procedures) nominated 149 differentially expressed genes (Fig 4) at a false-discovery rate (FDR) adjusted p-value < 0.05. 62 of 149 genes showed higher expression in the confluent monolayers while the remaining 87 of 149 genes had higher expression in the small islands (S2 Table). Among the genes with highest differential overexpression in the confluent monolayer were *TNNI3* (cardiac troponin 1 type 3), *MYL2* (cardiac myosin light chain 2), *ACTA1* (cardiac actin), *PDLIM3* (a structural protein expressed in *Z-*discs), and *TCAP* (telethonin, a regulator of sarcomere assembly at *Z*-discs). *MYH7* (cardiac myosin heavy chain β), a gene primarily expressed in the human ventricle, was also more highly expressed in the confluent monolayer. In contrast, *MYH6* (cardiac myosin heavy chain α) was expressed more in the smaller islands. This gene is expressed in atrial and ventricular myocytes during development but is lost in ventricular myocytes during maturation [45]. More broadly, the genes with the largest fractional overexpression in the small islands were extracellular matrix (ECM) and structural genes such as fibronectins and collagens. Gene Ontology analysis [37] revealed that genes increased transcriptionally in confluent monolayers were associated with myocardial contraction and mitochondrial function (Fig 4a and S2 and S3 Tables). Genes overexpressed in small islands were linked to extracellular matrix, cell adhesion and vascular formation.

To place these trends onto the timeline of cardiac development, we then compared these data with published RNA sequencing data from embryonic stem cells (ESCs), mesoderm (MES), cardiac progenitor cells (CP), embryonic stem cell-derived cardiomyocytes (ESC-CM), and mature adult cardiomyocytes (Adult-CM, Fig 4b). These samples have been described in Ref. [38]. Between the CP and Adult-CM, we identified 3,463 genes with significant differential expression (adjusted p-value < 0.05). We defined a vector in gene-expression space by the fold-change in expression for each of these genes, moving from CP to Adult-CM. We then used the gene expression vectors (log_2_ TPM) for each of the other samples and projected them onto the CP to Adult-CM axis. By this measure, there was a small but statistically significant difference between the small island and confluent samples: the confluent monolayers were only 4.7% ± 0.1% (mean ± s.e.m.) closer to Adult-CM than were the small islands (p = 5×10^−6^). Thus island size contributes little to global transcriptional maturation.

Surprisingly, a different picture emerged when we focused analysis on the 149 genes with significant differential expression between the confluent monolayers and small islands. Again we defined a vector in gene expression space by the fold-change in expression for each of these 149 genes, moving from CP to Adult-CM. For this subset of genes, the difference between the small islands and confluent monolayers accounted for 58% ± 18% (mean ± s.e.m.) of the difference between CP and Adult-CM (p = 2×10^−9^). Thus, for a subset of genes, island size drove the majority of the maturation that occurs during normal development. This observation suggests that cardiomyocytes contain a transcriptional network that is predominantly responsive to the local cellular environment and that other aspects of transcriptional maturation must be driven by distinct cues.

### Islands of different shapes elucidate mechanism of size-dependent maturation

With both optical electrophysiology and RNA sequencing showing significant differences between small and large islands, we next investigated the mechanism of island size-dependent maturation. We first tested whether the onset of beating depended on island size: more spontaneous activity in larger islands early in culture could lead to greater functional and transcriptional maturity later on. To test this hypothesis, we recorded movies of contractile motion as a function of island size and time post plating (S10 Fig, S3 Movie). All islands started beating at the same time, but at early stages the larger islands beat more slowly. Size-dependent differences in beat rate became insignificant by 12 dpp. Given the uniform onset of beating, the slower initial beat rate in larger islands, and the eventual convergence of the beat rates, we concluded that differences in spontaneous beating are unlikely to account for the greater maturity of cells in the larger islands.

**Fig 5:**
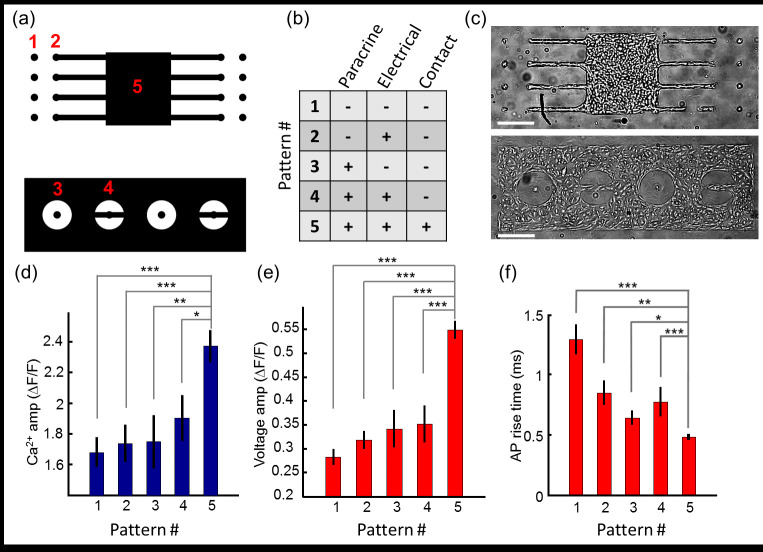
Growth on patterned islands identifies factors that influence hiPSC-CM maturation. a) Cells were cultured on patterned islands with five regions in which different intercellular influences were active. b) Chart showing the modes of intercellular interaction active in each region. c) Image of hiPSC-CM cultured on patterned islands. Scale bar 500 μm. d) Amplitude of calcium transient; e) Amplitude of voltage transient; f) Action potential rise time, for the five regions indicated in (a). In (d-f) measurements were performed on *n* = 282 samples. Error bars represent s.e.m. * p ≤ 0.05; ** p ≤ 0.01; *** p ≤ 0.001.

We thus considered three other mechanisms by which island size might influence maturation. 1) Cells might secrete soluble paracrine factors which accumulated to higher local concentrations around larger islands (“paracrine effect”). For secretion at a constant rate per unit area of island, one expects the concentration at the island surface to scale as *C* ∝ *L*/*D*, where *L* is the island edge length and *D* is the diffusion coefficient of the secreted factor [46]. Although a portion of any secreted factor might become uniformly distributed within a dish, experiments on different-sized islands in the same dish controlled for this background effect. To explore the role of paracrine effects, we compared small islands plated far from their neighbors (region 1 in Fig 5) to small islands separated by a thin gap from a bigger island (region 3). 2) Cells might interact through electrical signaling (e.g. changes in resting potential). We compared small isolated islands (region 1) to small islands of the same dimensions, coupled by a thin bridge to a large syncytium (region 2). Cells in region 2 beat synchronously and with undetectable delay relative to the parent syncytium, confirming the robustness of the electrotonic coupling (S11 Fig). Region 4 comprised small islands, with both paracrine and electrical coupling to a nearby large island. 3) Cells might interact through direct contact, e.g. via surface-bound receptors, gap junctions, or mechanical forces. The mean number of contact interactions per cell is proportional to the ratio of island perimeter to island area. Thus, cells in region 5 had stronger contact interactions, on average, than cells in regions 1–4. Fig 5b summarizes the qualitative effects that would be accentuated in each geometry.

Fig 5c shows representative images of hiPSC-CM seeded on the patterns from Fig 5a. We analyzed the voltage and Ca^2+^ waveforms as above to assess the functional maturity of hiPSC-CM in each geometry (Fig 5d–5f). Neither electrical coupling to a large syncytium (region 2, 3), nor diffusional coupling to a large syncytium (region 3, 4), produced strong effects on action potential waveform parameters. In these four geometries, the functional maturation was only modestly greater than in an isolated small island (region 1).

The greatest change in action potential waveform parameters was observed among cells embedded in a large syncytium (region 5). This geometry was the only one tested in which the majority of cells were in direct mechanical contact with neighbors on all sides. Therefore, we conclude that cells grown in larger islands matured primarily through short-range interactions with neighboring cells. The present results cannot distinguish whether these short-range interactions are mechanical or biochemical; but they rule out electrical coupling or long-rage paracrine signaling as dominant contributors to the geometry-dependent changes in voltage and Ca^2+^ waveforms. Examination of many islands immunostained with a marker for cardiac troponin T revealed more myofibril banding in cells in the interior of the islands relative to cells at the edges (S12 Fig). These observations support the importance of nearest-neighbor contacts in driving maturation.

## Discussion

High-throughput optical electrophysiology and gene expression profiling revealed that functional maturity of hiPSC-CM grown in confluent islands increased as a function of island size. For a subset of genes associated with functional maturation, changes in island size accounted for approximately 58% of the change in gene expression that occurs during cardiac maturation in human development. This observation points to a precise transcriptional network whose expression level is strongly regulated by nearest-neighbor contacts. By varying the geometry of the islands, we determined that short-range contact interactions played the dominant role in governing the observed maturation, rather than electrical coupling, pacing, or diffusible paracrine factors.

The nature of these short-range interactions remains to be determined. Gene ontology analysis of the differentially expressed genes revealed that the four terms most represented in the smaller islands were related to extracellular components (S2 Table). The presence of a differentially expressed extracellular component does not indicate the range over which this component acts—whether it is bound locally to the extracellular matrix or whether it is free to diffuse to distant cells. Thus the differential expression of secreted proteins in the smaller islands is not necessarily at odds with the observation that the island size-dependent maturational cues seem to be short range.

In an absolute sense, growth in islands of different sizes led to only modest changes in maturation. This was true morphologically, functionally, and transcriptionally. At the morphological level, the cells did not elongate, develop T-tubules, or show clear sarcomeric Z-disks. At the functional level, the persistence of spontaneous activity indicated an immature electrophysiological state, and the cells never developed the rapid upstroke observed in mature CM. The transcriptional changes were the only modality that could be placed quantitatively on a developmental axis. Considering all genes that changed from cardiac progenitors to Adult-CM, the hiPSC-CM resided about half way along the trajectory; but cells grown on large islands were only 4.7% closer to adult CM than were cells grown on small islands.

The importance of short-range interactions highlights a limitation of the planar cell culture geometry. The cells were relatively flat, and only touched at their edges. Thus the area of cell-cell contact for a cell completely surrounded by other cells remained a minuscule fraction of the total cell surface area. Clearly a three-dimensional geometry would provide dramatically more cell-cell contact[47]. Indeed cells grown in 3D cultures have shown improved alignment, hypertrophy, and replication[48]. Human embryonic stem cell-derived CMs grown in 3D patches also showed faster conduction velocity, longer sarcomeres, and upregulated expression of genes involved in cardiac contraction[49]. However, unvascularized 3-D cell culture encounters difficulties with adequate oxygen delivery to internal cells. The spacing of the capillary bed in adult human myocardium is approximately 20 μm [50], suggesting that any 3-D CM culture more than a few cells thick will produce hypoxia in the center. Coculture of hiPSC-CM with vascular endothelial and stromal cells is a promising approach to resolve this mass-transport problem[48], though actively perfusing the nascent vessels *in vitro* remains a challenge.

Mechanical and electrical pacing further contributes to enhanced maturation, particularly in 3D cultures[15]. It is natural to assume that these stimuli, mimicking the cardiac milieu, somehow directly promote maturation. However, a recent study suggests that an additional function of dynamic pacing is to improve mass transport by stirring the local medium.[51] This example highlights the difficulty of assigning changes in maturation to single causes.

Several groups have shown increased maturation by growing hiPSC-CM on soft substrates[52,53] or in elastomeric gels [48]. However, the mechanical micro-environment likely accounts for only a portion of the short-range maturational cues. The cardiac extracellular matrix (ECM) is a complex assembly of glycosaminoglycans, glycoproteins, and adhesive proteoglycans that have both signaling and mechanical functions [54]. Cardiomyocytes grown on decellularized heart ECM showed greater maturation as measured by protein expression than cells grown on mechanically similar liver ECM [55]. Recent efforts to grow cardiomyocytes in gel micro-capsules provides an intriguing way to create a cardiac-like mechanical and biochemical environment while preserving oxygenation [56]. Cell culture geometries in which secreted macromolecular components are retained while oxygen and small molecules can readily interchange with a reservoir are promising for future tissue engineering applications.

Planar cell culture offers many advantages from the perspective of convenience and ease of imaging. Thus it is useful to contemplate whether the advantages of micro-capsule culture can be obtained in a planar geometry. One plausible approach is to culture cells between thin semi-permeable sheets that retain macromolecules but pass nutrients and oxygen.

The combination of optical electrophysiology and transcriptional profiling will provide a rich assessment of how each aspect of cell culture contributes to multiple distinct measures of functional maturation. The results presented here have focused on the effect of local geometry on maturation, but clearly the tools of optical electrophysiology open the possibility of rapid, accurate functional phenotyping of human cardiomyocytes in a wide range of conditions relevant to health and disease.

## Supporting information

S1 Fig Procedure for growing hiPSC-CM on patterned elastomeric substrates. A) Synthesis procedure for constructing patterns of covalently bound fibronectin atop a soft polyacrylamide substrate. B) Image of fluorescently labeled fibronectin showing the fidelity of the stamping pattern. Image is 5 mm across. C) Transmitted light image of HEK293 cells grown on the patterned fibronectin. The defects in the fibronectin pattern in S1b Fig. (e.g. the circular region missing in the lower left large square) are faithfully reproduced in the pattern of cell growth.
(PNG)

S2 Fig Comparison of AP waveforms in hiPSC-CM, as measured by the genetically encoded voltage indicator QuasAr2 and the voltage-sensitive dye, FluoVolt. Top: Simultaneous dual-wavelength measurements in the same hiPSC-CM. QuasAr2 was excited at 637 nm with fluorescence detection at 720 nm. FluoVolt was excited at 488 nm with fluorescence detection at 525 nm. Bottom: effect of QuasAr2 expression on AP waveform. Fluovolt fluorescence was recorded from two neighboring cells, one expressing QuasAr2 and one not expressing QuasAr2. There was no detectable difference in the AP waveforms attributable to expression of QuasAr2.
(PNG)

S3 Fig Image analysis pipeline. (A) Raw QuasAr2 image from one field of view with island size 250 μm. (B) Background image, calculated by performing an image opening operation. The erosion/dilation element was elliptical, with width slightly larger than the island size and height 200 μm. (C) Image after background subtraction. Images were then masked using an absolute threshold for the entire data set, so all pixels where there was no expressed protein were excluded from further analysis. (D) Islands were automatically selected based on array size, and islands that were incomplete or otherwise had a bad morphology were excluded. The island with the yellow star is used in (E)-(I). (E) Fluorescence time traces for voltage (red) and calcium (blue) calculated by taking the flat average of all pixels in the pink box in (D). Baseline drift (black) was calculated by interpolating between minimum points with a sliding window whose width was slightly longer than the beat period. The baseline drift (black trace) was subtracted from the recordings. Each trace was then divided by baseline to calculate Δ*F*/*F*_0_ traces, shown in (I). (F) Time-averaged fluorescence of the selected island. (G) Equal-time cross-correlation of whole-island intensity (shown in (I)) with intensity-trace at each pixel, highlighting pixels with synchronized voltage activity. (H) Sensitivity image calculated by dividing (G) by (F), showing Δ*F*/*F*_0_ for each pixel in the island, masked to reject pixels identified as background. The distribution of these pixel sensitivities, pooled across all islands of the same size, are used calculate voltage and calcium amplitudes. (I) Beats were automatically identified by an edge-finding algorithm, and action potential widths were calculated at defined thresholds. The rise rate was calculated from the maximum derivative on each rising edge.
(PNG)

S4 Fig Automated parameterization of the data shown in Fig 2b & 2d. A) Voltage-imaging data. The green lines indicate the timing of the upstroke, and the width of the action potential at 30%, 50%, and 70% of return to baseline. B) Calcium imaging data. The purple lines indicate the amplitude of the calcium transient, and the width of the calcium transient at 30%, 50%, and 70% of return to baseline.
(PNG)

S5 Fig Automated parameterization of voltage imaging traces from islands of size A) 50 μm, B) 100 μm, C) 500 μm, and D) 1000 μm. (PNG)

S6 Fig Island size-dependent maturation. (A) Histograms of calcium transient amplitudes for every pixel (see S2H Fig), pooled for each island size. The number of pixels in the histogram is indicated in the upper right of each pane. The black line indicates the mean, with fluorescent but unresponsive pixels (Δ*F*/*F*0 < 0.2) excluded from the average. (B) Histograms of AP voltage amplitude. The black line is the average. (C) Histogram of rate of voltage rise. The number of islands in the histogram is indicated in the upper right of each pane and the black line is the mean of all islands.
(PNG)

S7 Fig Subdividing large islands to create synthetic small islands as a check for image analysis artifacts. Calculations parallel those in S5 Fig where trends were derived from the full data set. (A) Histograms of calcium transient amplitudes for every pixel, pooled from subdivisions of a 1 mm island to smaller synthetic islands. The number of pixels in the histogram is indicated in the upper right of each pane. The black line indicates the mean, with fluorescent but unresponsive pixels (Δ*F*/*F*_0_ < 0.2) excluded from the average. The red, blue and green lines are the mode, median and 90% threshold, respectively. (B) Histograms of AP voltage amplitude. (C) The voltage rise rate histogram with rates measured for each island. The number of islands (subdivision from one 1 mm island size) in the histogram is indicated in the upper right of each pane and the black line is the mean of all islands.
(PNG)

S8 Fig Conduction velocity of confluent hiPSC-CM cultures. HiPSC-CM were seeded in a uniform syncytium and paced just to the left of the field of view. The voltage wave propagated from left to right. (A) QuasAr2 fluorescence and subdivided ROIs. (B) Overlaid time traces from each ROI, with an expanded view of one beat. (C) Mean AP rise time (50% of max) as a function of ROI coordinate. The fit shows the conduction velocity.
(PNG)

S9 Fig Simulations of action potential waveforms in the Aliev-Panfilov model. A) Simulations of AP waveforms of single cells with heterogeneous intrinsic beat rates, achieved by assigning to each cell a leakage current selected from a Gaussian distribution with 22% coefficient of variation. B) Simulation of the action potential waveform in a 16x16 array of coupled cells. The coupled syncytium adopted the mean beat rate of the constituent cells.
(PNG)

S10 Fig Spontaneous beat rate as a function of days post plating and island size. Initially the smaller islands had a higher spontaneous beat rate, but by 12 dpp this difference had disappeared and all island sizes showed a similar spontaneous beat rate. Error bars represent s.e.m.
(PNG)

S11 Fig Strong electrotonic coupling across thin bridges of hiPSC-CM. The plot compares simultaneously measured QuasAr2 fluorescence traces in regions 2 and 5 of the crab-shaped pattern snown in the top of Fig 4a. The fluorescence recordings have been scaled vertically to facilitate comparison. The upstroke is synchronous in the two regions to within measurement precision, indicating strong electrotonic coupling.
(PNG)

S12 Fig Sarcomere organization as revealed by cardiac troponin T immunostaining. The island size is indicated in each panel. In the 500 μm and 1000 μm islands, banding, which is present in mature cells *in vivo*, is clear in the interior but is absent on the periphery (left edge of images). In the 50 μm and 100 μm islands, where most cells are on the perimeter, less banding is present.
(PNG)

S1 Movie Fluorescence imaging of the QuasAr2 voltage indicator in 250 μm islands of hiPSC-CM. The islands all showed asynchronous spontaneous beating.
(MP4)

S2 Movie Fluorescence imaging of the GCaMP6f Ca^2+^ indicator in 250 μm islands of hiPSC-CM. The islands all showed asynchronous spontaneous beating.
(MP4)

S3 Movie Transmitted light image of a large set of patterned islands containing beating hiPSC-CM. (MP4)

S1 Table Complete transcriptomic data for all genes measured in small and large islands. (XLSX)

S2 Table Genes differentially expressed between hiPSC-CM grown on small and large islands. (XLSX)

S3 Table Gene Ontology analysis of genes differentially expressed between hiPSC-CM grown on small and large islands. (XLSX)
