# Supplementary figures and images for: Geometry-dependent functional changes in iPSC-derived cardiomyocytes probed by functional imaging and RNA sequencing
Source: PLoS One. 2017 Mar 23;12(3):e0172671. doi: 10.1371/journal.pone.0172671 (PMC5363803; doi:10.1371/journal.pone.0172671)

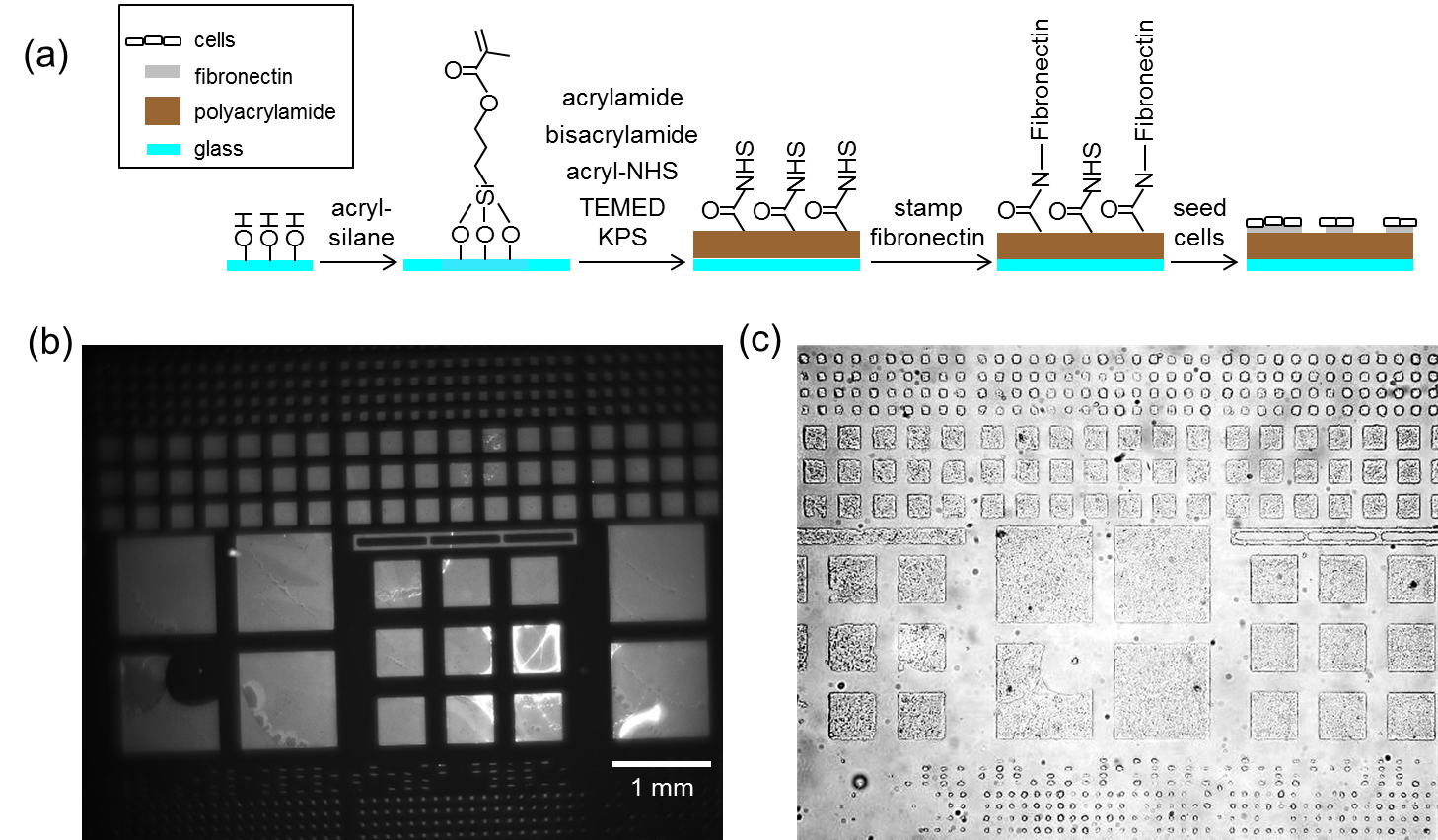

Supplement: S1 Fig — A) Synthesis procedure for constructing patterns of covalently bound fibronectin atop a soft polyacrylamide substrate. B) Image of fluorescently labeled fibronectin showing the fidelity of the stamping pattern. Image is 5 mm across. C) Transmitted light image of HEK293 cells grown on the patterned fibronectin. The defects in the fibronectin pattern in S1b Fig. (e.g. the circular region missing in the lower left large square) are faithfully reproduced in the pattern of cell growth. (PNG) [file pone.0172671.s001.png]

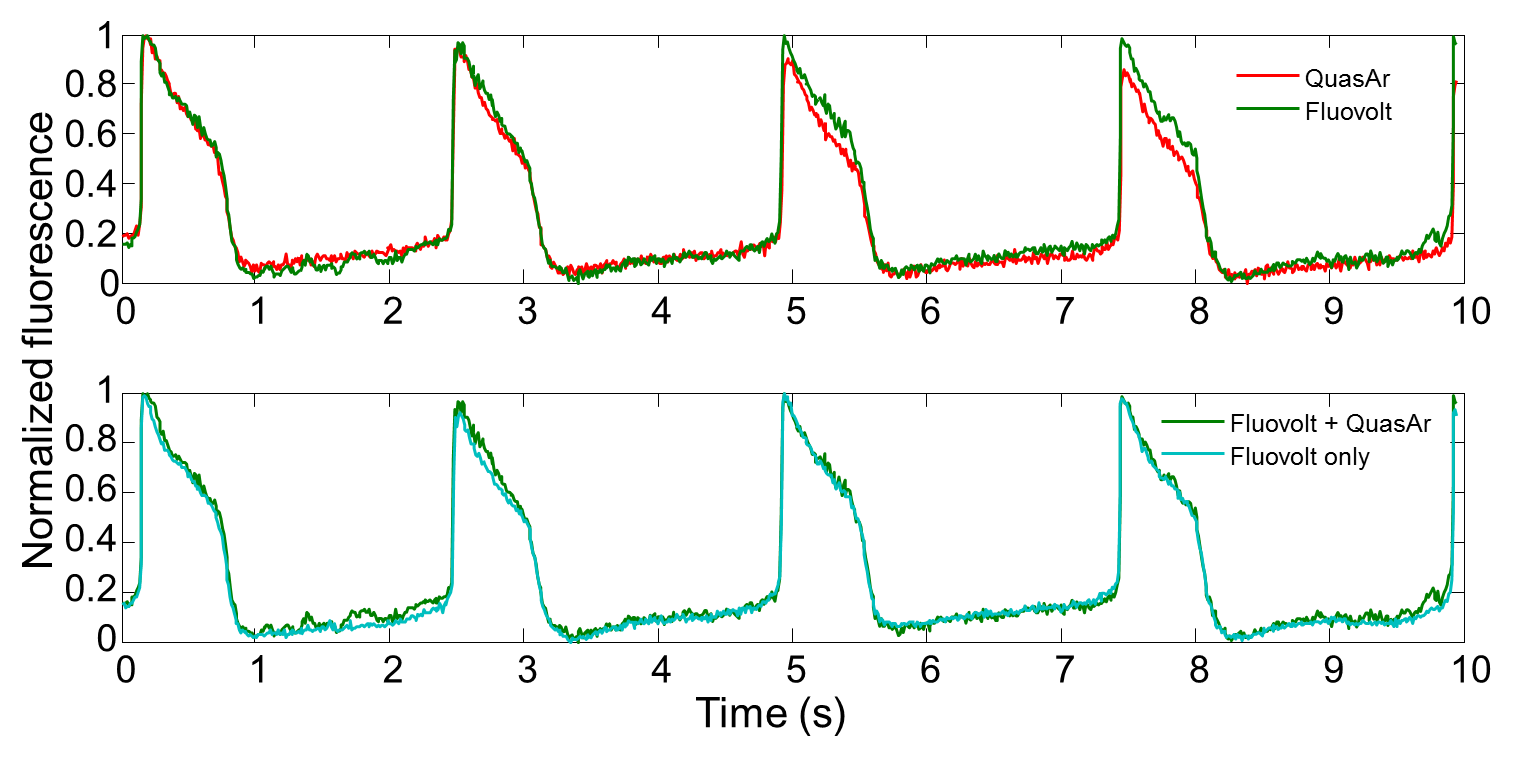

Supplement: S2 Fig — Top: Simultaneous dual-wavelength measurements in the same hiPSC-CM. QuasAr2 was excited at 637 nm with fluorescence detection at 720 nm. FluoVolt was excited at 488 nm with fluorescence detection at 525 nm. Bottom: effect of QuasAr2 expression on AP waveform. Fluovolt fluorescence was recorded from two neighboring cells, one expressing QuasAr2 and one not expressing QuasAr2. There was no detectable difference in the AP waveforms attributable to expression of QuasAr2. (PNG) [file pone.0172671.s002.png]

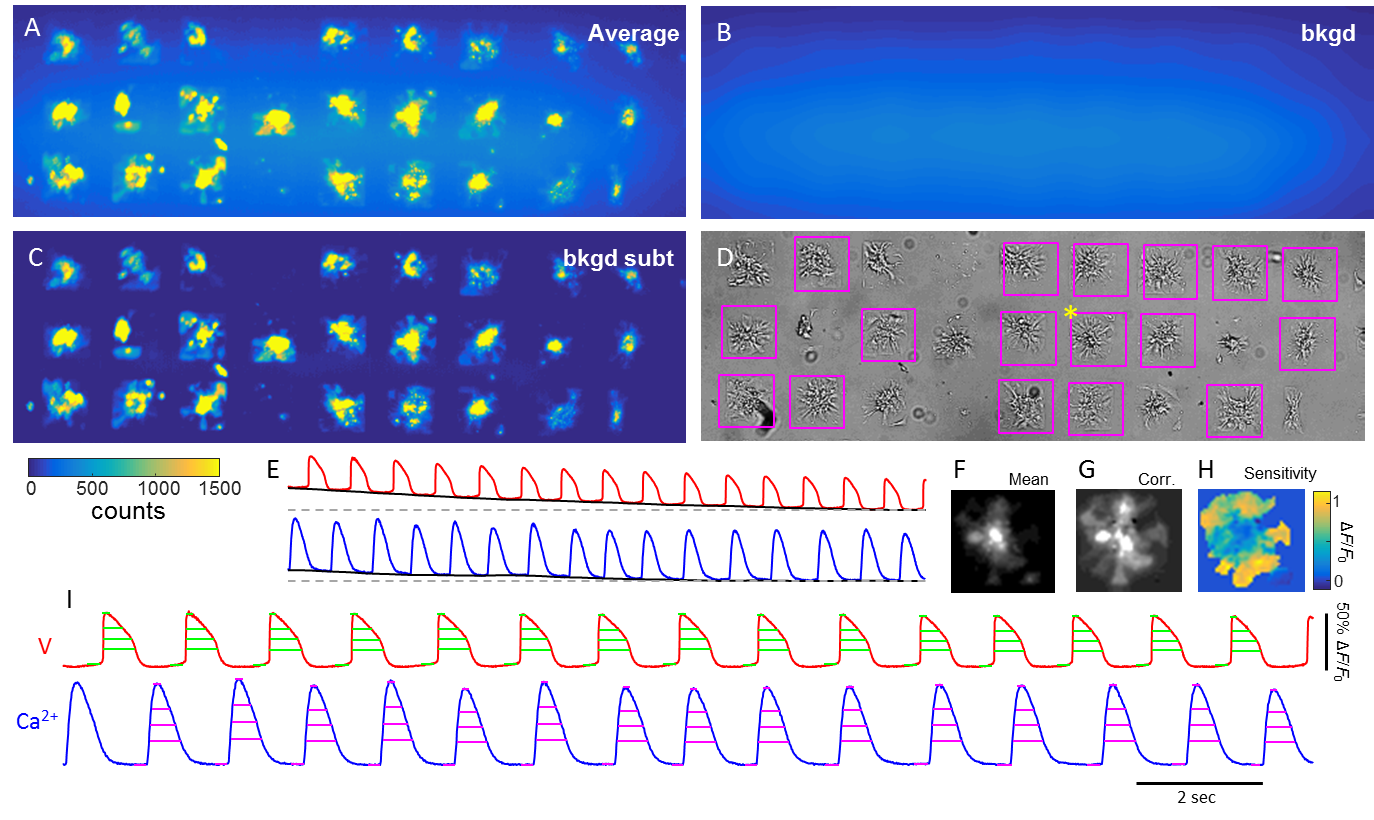

Supplement: S3 Fig — (A) Raw QuasAr2 image from one field of view with island size 250 μm. (B) Background image, calculated by performing an image opening operation. The erosion/dilation element was elliptical, with width slightly larger than the island size and height 200 μm. (C) Image after background subtraction. Images were then masked using an absolute threshold for the entire data set, so all pixels where there was no expressed protein were excluded from further analysis. (D) Islands were automatically selected based on array size, and islands that were incomplete or otherwise had a bad morphology were excluded. The island with the yellow star is used in (E)-(I). (E) Fluorescence time traces for voltage (red) and calcium (blue) calculated by taking the flat average of all pixels in the pink box in (D). Baseline drift (black) was calculated by interpolating between minimum points with a sliding window whose width was slightly longer than the beat period. The baseline drift (black trace) was subtracted from the recordings. Each trace was then divided by baseline to calculate ΔF/F0 traces, shown in (I). (F) Time-averaged fluorescence of the selected island. (G) Equal-time cross-correlation of whole-island intensity (shown in (I)) with intensity-trace at each pixel, highlighting pixels with synchronized voltage activity. (H) Sensitivity image calculated by dividing (G) by (F), showing ΔF/F0 for each pixel in the island, masked to reject pixels identified as background. The distribution of these pixel sensitivities, pooled across all islands of the same size, are used calculate voltage and calcium amplitudes. (I) Beats were automatically identified by an edge-finding algorithm, and action potential widths were calculated at defined thresholds. The rise rate was calculated from the maximum derivative on each rising edge. (PNG) [file pone.0172671.s003.png]

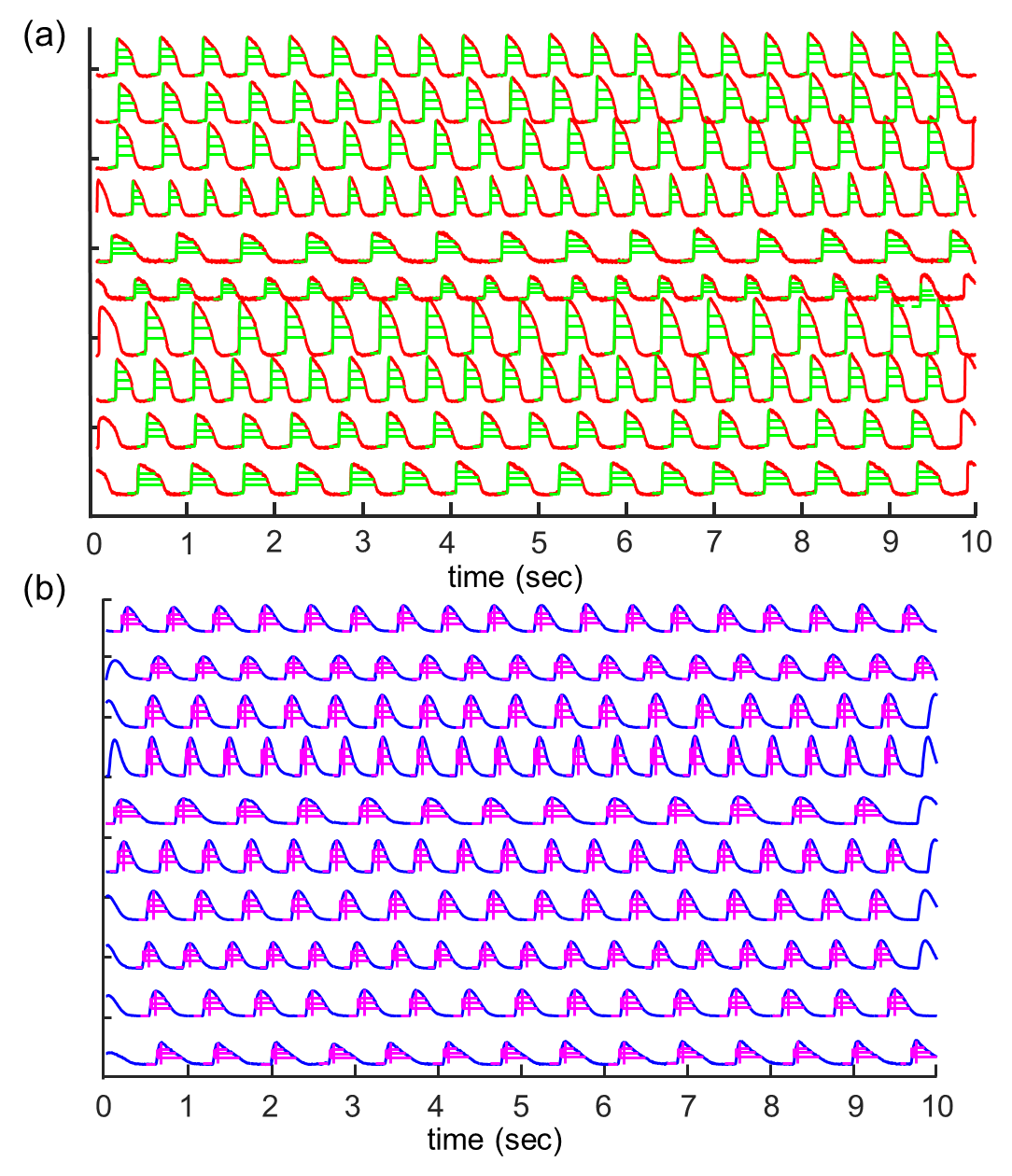

Supplement: S4 Fig — A) Voltage-imaging data. The green lines indicate the timing of the upstroke, and the width of the action potential at 30%, 50%, and 70% of return to baseline. B) Calcium imaging data. The purple lines indicate the amplitude of the calcium transient, and the width of the calcium transient at 30%, 50%, and 70% of return to baseline. (PNG) [file pone.0172671.s004.png]

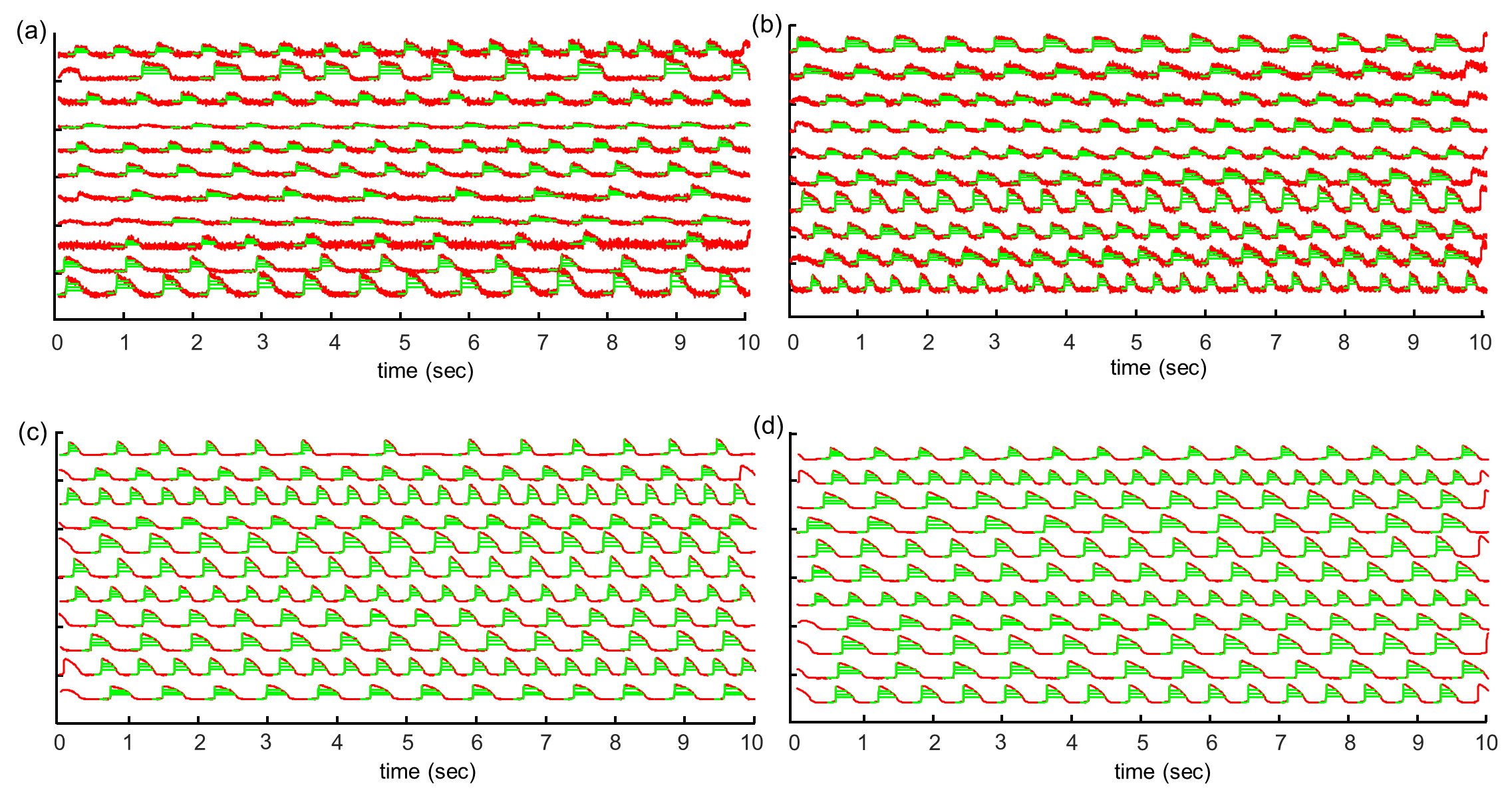

Supplement: S5 Fig — (PNG) [file pone.0172671.s005.png]

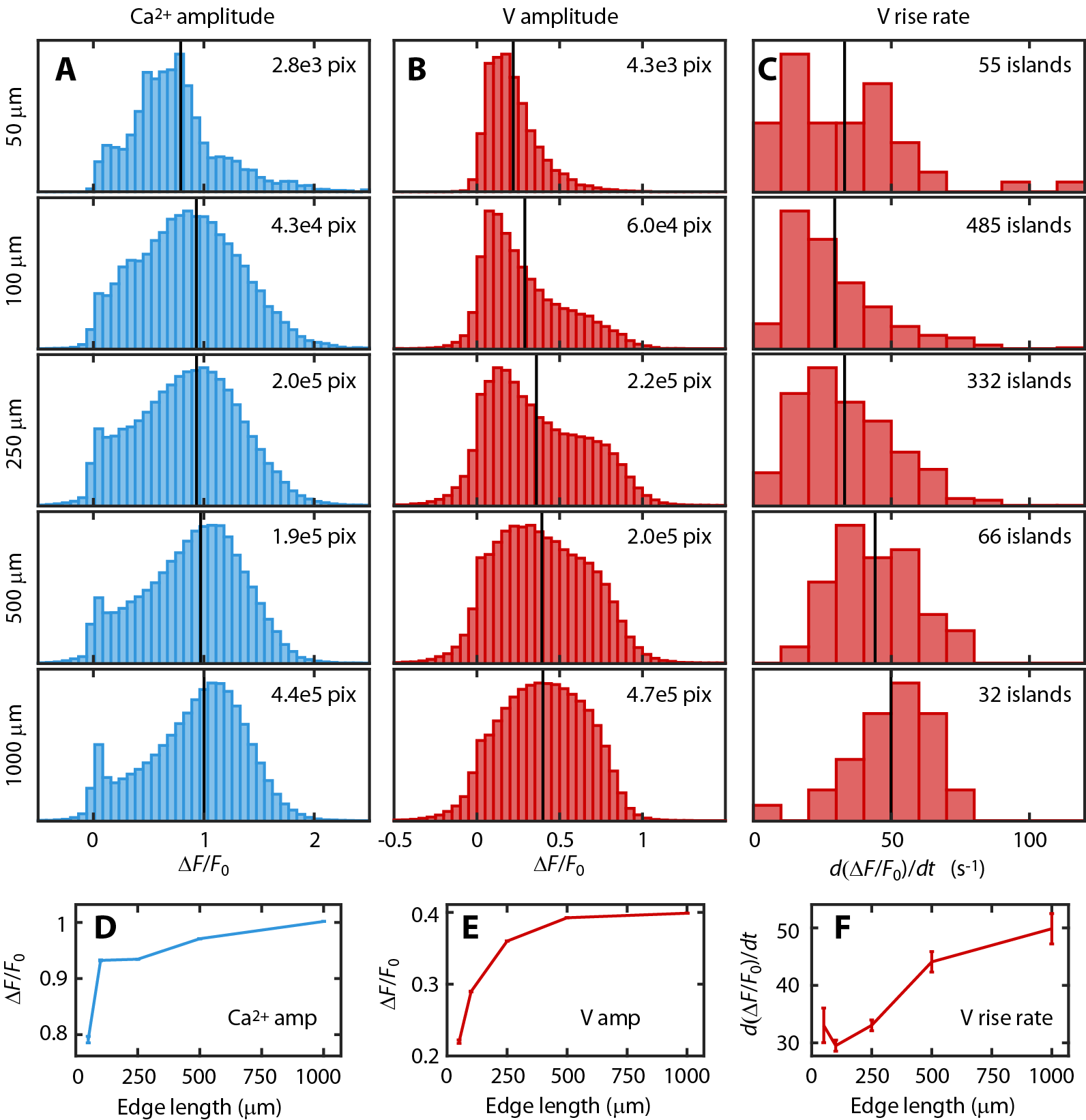

Supplement: S6 Fig — (A) Histograms of calcium transient amplitudes for every pixel (see S2H Fig), pooled for each island size. The number of pixels in the histogram is indicated in the upper right of each pane. The black line indicates the mean, with fluorescent but unresponsive pixels (ΔF/F0 < 0.2) excluded from the average. (B) Histograms of AP voltage amplitude. The black line is the average. (C) Histogram of rate of voltage rise. The number of islands in the histogram is indicated in the upper right of each pane and the black line is the mean of all islands. (PNG) [file pone.0172671.s006.png]

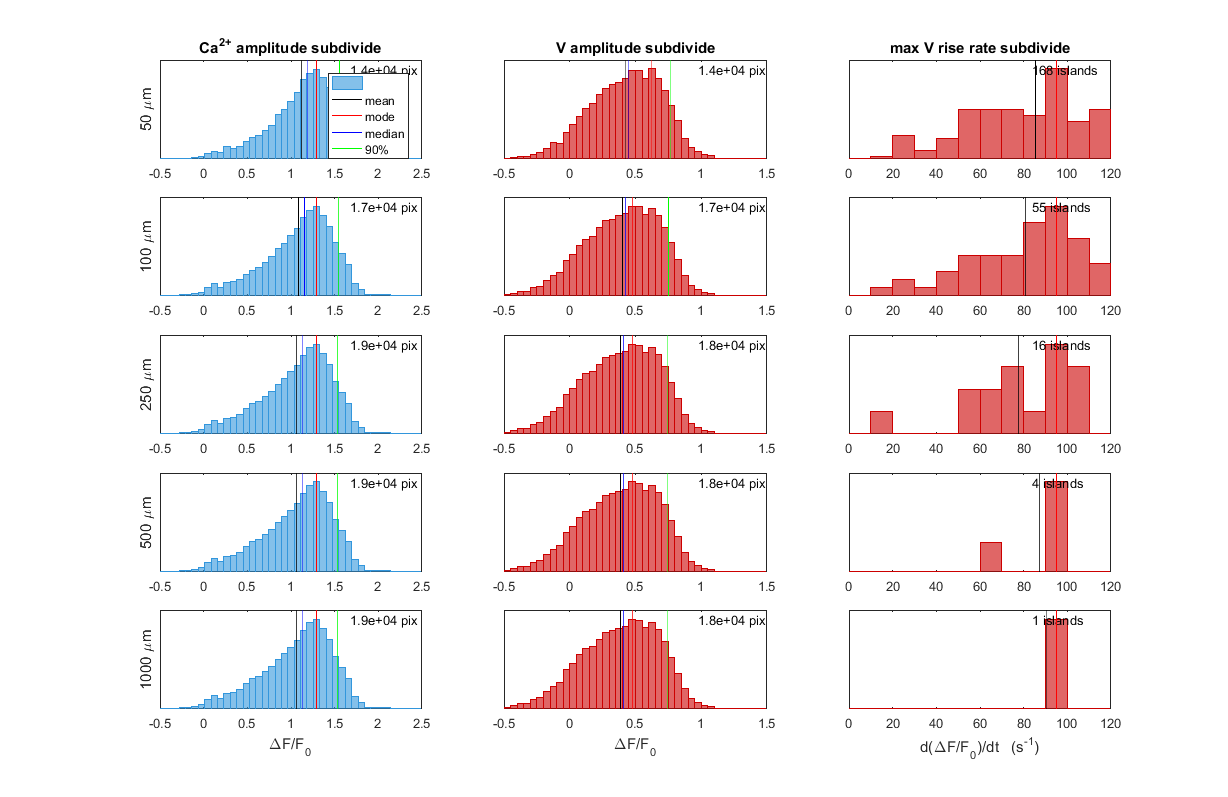

Supplement: S7 Fig — Calculations parallel those in S5 Fig where trends were derived from the full data set. (A) Histograms of calcium transient amplitudes for every pixel, pooled from subdivisions of a 1 mm island to smaller synthetic islands. The number of pixels in the histogram is indicated in the upper right of each pane. The black line indicates the mean, with fluorescent but unresponsive pixels (ΔF/F0 < 0.2) excluded from the average. The red, blue and green lines are the mode, median and 90% threshold, respectively. (B) Histograms of AP voltage amplitude. (C) The voltage rise rate histogram with rates measured for each island. The number of islands (subdivision from one 1 mm island size) in the histogram is indicated in the upper right of each pane and the black line is the mean of all islands. (PNG) [file pone.0172671.s007.png]

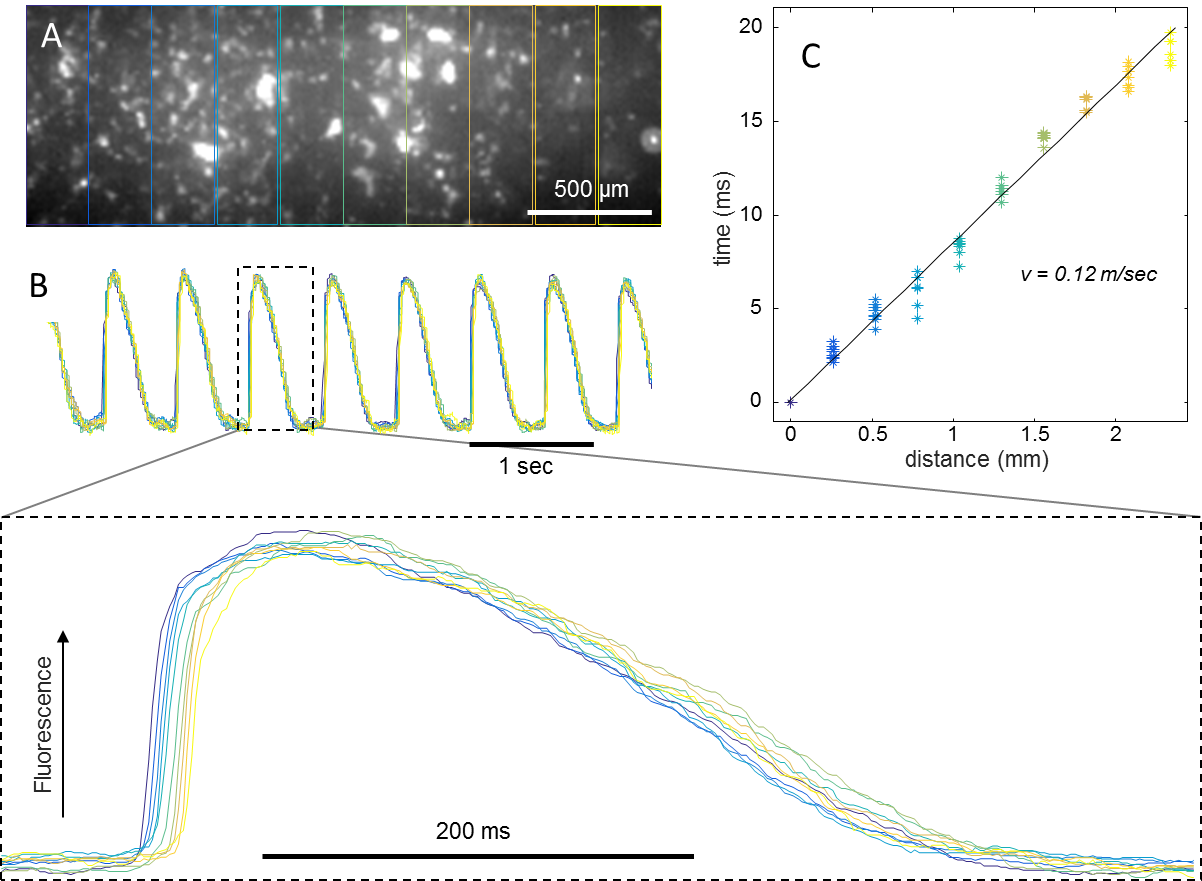

Supplement: S8 Fig — HiPSC-CM were seeded in a uniform syncytium and paced just to the left of the field of view. The voltage wave propagated from left to right. (A) QuasAr2 fluorescence and subdivided ROIs. (B) Overlaid time traces from each ROI, with an expanded view of one beat. (C) Mean AP rise time (50% of max) as a function of ROI coordinate. The fit shows the conduction velocity. (PNG) [file pone.0172671.s008.png]

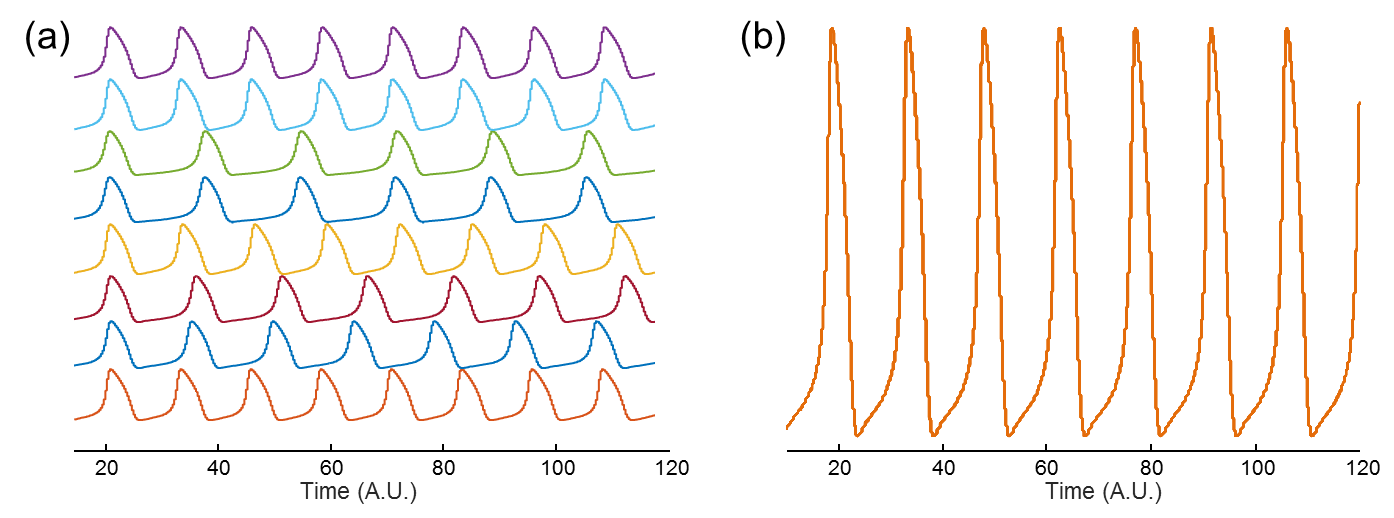

Supplement: S9 Fig — A) Simulations of AP waveforms of single cells with heterogeneous intrinsic beat rates, achieved by assigning to each cell a leakage current selected from a Gaussian distribution with 22% coefficient of variation. B) Simulation of the action potential waveform in a 16x16 array of coupled cells. The coupled syncytium adopted the mean beat rate of the constituent cells. (PNG) [file pone.0172671.s009.png]

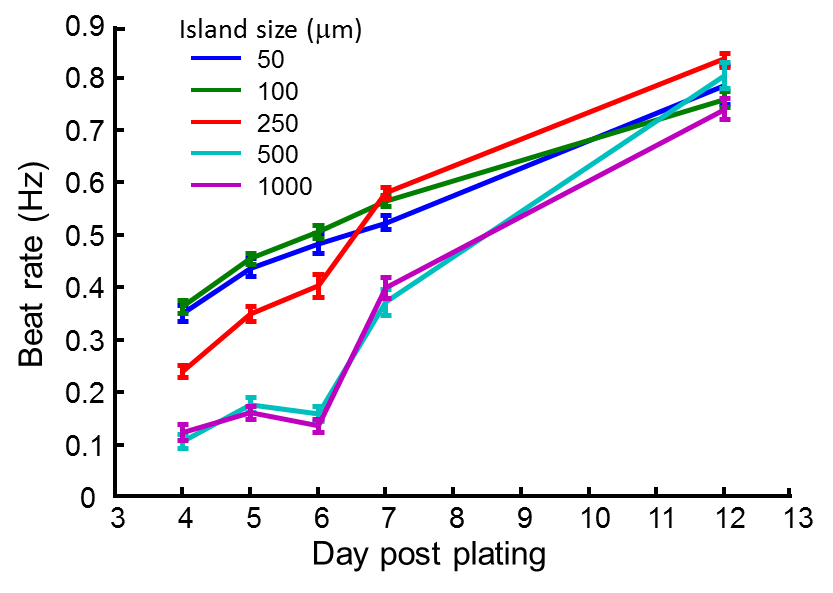

Supplement: S10 Fig — Initially the smaller islands had a higher spontaneous beat rate, but by 12 dpp this difference had disappeared and all island sizes showed a similar spontaneous beat rate. Error bars represent s.e.m. (PNG) [file pone.0172671.s010.png]

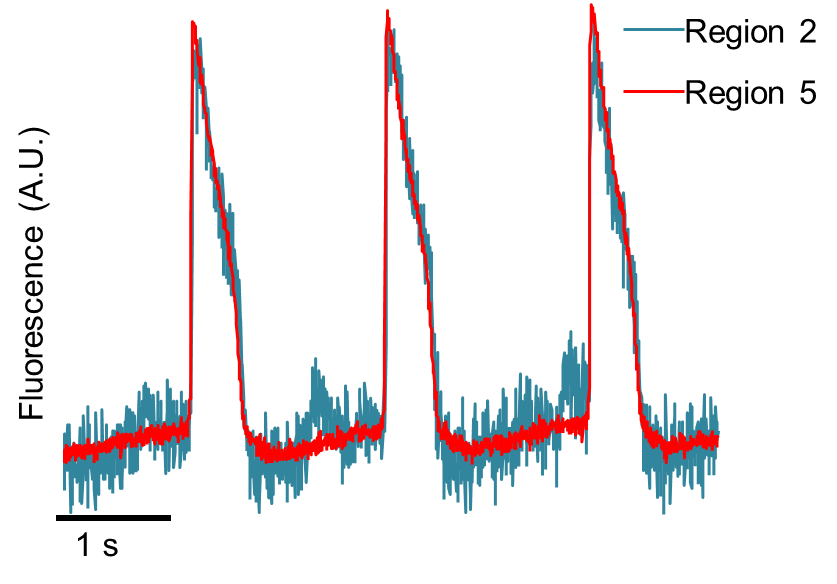

Supplement: S11 Fig — The plot compares simultaneously measured QuasAr2 fluorescence traces in regions 2 and 5 of the crab-shaped pattern snown in the top of Fig 4a. The fluorescence recordings have been scaled vertically to facilitate comparison. The upstroke is synchronous in the two regions to within measurement precision, indicating strong electrotonic coupling. (PNG) [file pone.0172671.s011.png]

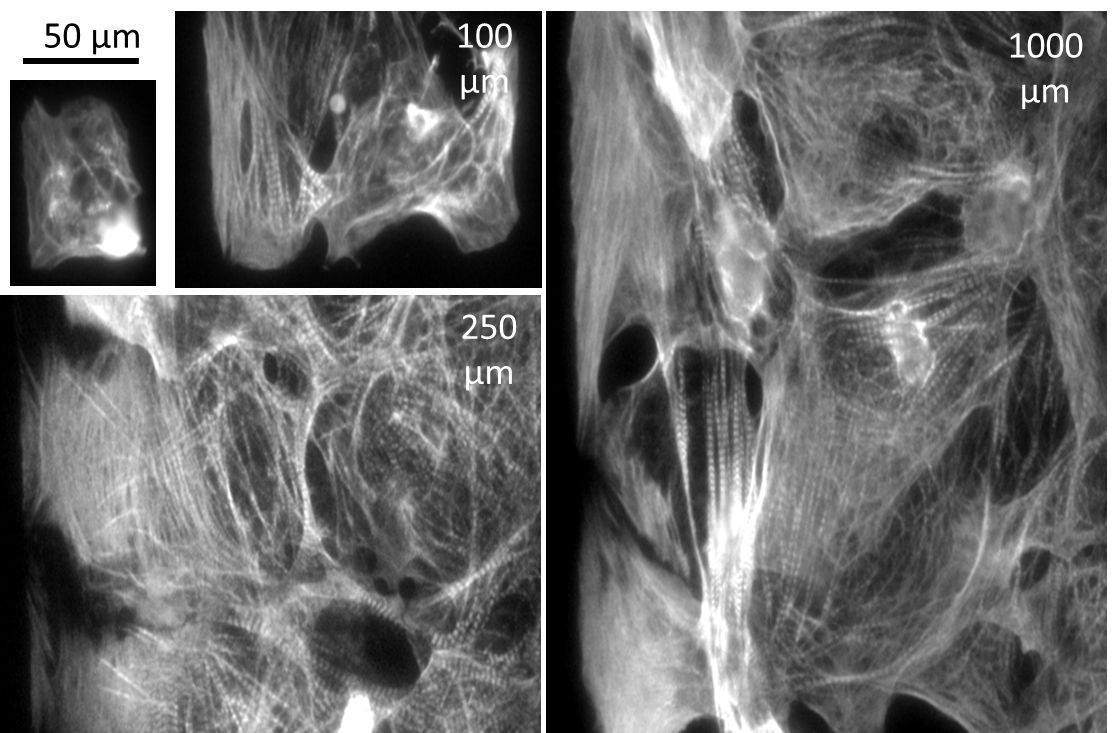

Supplement: S12 Fig — The island size is indicated in each panel. In the 500 μm and 1000 μm islands, banding, which is present in mature cells in vivo, is clear in the interior but is absent on the periphery (left edge of images). In the 50 μm and 100 μm islands, where most cells are on the perimeter, less banding is present. (PNG) [file pone.0172671.s012.png]
